# Identification of anoikis-related gene signatures and construction of the prognosis model in prostate cancer

**DOI:** 10.3389/fphar.2024.1383304

**Published:** 2024-06-18

**Authors:** Wanying Kang, Chen Ye, Yunyun Yang, Yan-Ru Lou, Mingyi Zhao, Zhuo Wang, Yuan Gao

**Affiliations:** ^1^ School of Pharmacy, Fudan University, Shanghai, China; ^2^ Life Science and Biopharmaceutical College, Shenyang Pharmaceutical University, Shenyang, China; ^3^ Changhai Hospital, Second Military Medical University, Shanghai, China

**Keywords:** prostate cancer, anoikis-related genes, biochemical recurrence, immune infiltration, prognosis

## Abstract

**Background:**

One of the primary reasons for tumor invasion and metastasis is anoikis resistance. Biochemical recurrence (BCR) of prostate cancer (PCa) serves as a harbinger of its distant metastasis. However, the role of anoikis in PCa biochemical recurrence has not been fully elucidated.

**Methods:**

Differential expression analysis was used to identify anoikis-related genes based on the TCGA and GeneCards databases. Prognostic models were constructed utilizing LASSO regression, univariate and multivariate Cox regression analyses. Moreover, Gene Expression Omnibus datasets (GSE70770 and GSE46602) were applied as validation cohorts. Gene Ontology, KEGG and GSVA were utilized to explore biological pathways and molecular mechanisms. Further, immune profiles were assessed using CIBERSORT, ssGSEA, and TIDE, while anti-cancer drugs sensitivity was analyzed by GDSC database. In addition, gene expressions in the model were examined using online databases (Human Protein Atlas and Tumor Immune Single-Cell Hub).

**Results:**

113 differentially expressed anoikis-related genes were found. Four genes (EEF1A2, RET, FOSL1, PCA3) were selected for constructing a prognostic model. Using the findings from the Cox regression analysis, we grouped patients into groups of high and low risk. The high-risk group exhibited a poorer prognosis, with a maximum AUC of 0.897. Moreover, larger percentage of immune infiltration of memory B cells, CD8 Tcells, neutrophils, and M1 macrophages were observed in the high-risk group than those in the low-risk group, whereas the percentage of activated mast cells and dendritic cells in the high-risk group were lower. An increased TIDE score was founded in the high-risk group, suggesting reduced effectiveness of ICI therapy. Additionally, the IC50 results for chemotherapy drugs indicated that the low-risk group was more sensitive to most of the drugs. Finally, the genes EEF1A2, RET, and FOSL1 were expressed in PCa cases based on HPA website. The TISCH database suggested that these four ARGs might contribute to the tumor microenvironment of PCa.

**Conclusion:**

We created a risk model utilizing four ARGs that effectively predicts the risk of BCR in PCa patients. This study lays the groundwork for risk stratification and predicting survival outcomes in PCa patients with BCR.

## 1 Introduction

Prostate cancer (PCa) is ranked as the most common cancer among men worldwide, representing a considerable percentage of cancer-related deaths in the male demographic (Sung et al., 2021). According to estimations, PCa account for 29% of newly diagnosed male malignancies in the United States in 2023. In China, the occurrence of PCa has been on the rise with the aging population and increased screening rates (He et al., 2022; Siegel et al., 2023). Despite advancements in diagnostic and treatment options, around 30% of PCa patients who have radical prostatectomy developed biochemical recurrence (BCR) within a decade, with two-thirds of these recurrences manifesting within the first 2 years after surgery (Lin et al., 2019). A rise in serum prostate-specific antigen (PSA) levels after radical prostatectomy or radical radiotherapy signals biochemical recurrence. This increase was typically an early indicator of PCa progression, which could lead to PCa specific mortality or distant metastasis (Pound et al., 1999; Dess et al., 2017). For patients with BCR requiring salvage therapy, treatment options were limited (Wang M. et al., 2022). Therefore, identifying reliable prognostic biomarkers and understanding the underlying molecular mechanisms involved in BCR were crucial for better risk stratification and personalized treatment strategies in PCa.

Past studies have shown that some molecular prognostic biomarkers other than anoikis may also predict the risk of BCR of PCa. MiRNA such as miR-320a, miR-125A, and miR-196a were reported to be used for predicting the recurrence of PCa after radical prostatectomy (Pashaei et al., 2017; Zhan et al., 2020; Konoshenko and Laktionov, 2021). Moreover, the expression levels of mRNAs such as SAMD5 (Li et al., 2019) and ZNF154 (Zhang et al., 2018) served as prognostic biomarkers for predicting the risk of BCR in PCa. Furthermore, seven immune-related genes including (PPARGC1A、AKR1C2、COMP、EEF1A2、IRF5、NTM and TPX2) were identified as prognostic markers, showing an association with BCR-free survival in PCa patients (Lv et al., 2021). In addition, Prognostic subtypes established using senescence-associated lncRNAs were also closely associated with BCR-free survival in PCa (Feng et al., 2023). However, these molecular prognostic biomarkers above have not been validated in clinical application. In addition, there were some clinicopathological markers such as the Gleason score, clinical stage and PSA levels which were also applied to predict BCR following local PCa treatment (Cornford et al., 2017; D’Andrea et al., 2018). Nevertheless, various clinical outcomes were observed in these patients with the same biomarker (Li et al., 2019). Therefore, more sensitive BCR-related biomarkers for prediction should be developed.

Anoikis is a distinct type of programmed cell death that occurs when cells detach from the extracellular matrix or neighboring cells (Sattari Fard et al., 2023). This process is primarily initiated through the interplay of intrinsic and extrinsic pathways (Paoli et al., 2013). Anoikis is also a critical mechanism in maintaining tissue homeostasis by eliminating cells that have lost their anchorage, preventing their abnormal accumulation, and preventing metastasis (Taddei et al., 2012). Dysfunctions in anoikis can facilitate tumor invasion and migration, the establishment of metastasis in distant tissues, and the development of drug resistance (Kim et al., 2021). Moreover, tumor cells are able to resist anoikis and survive by utilizing a variety of ways, including EMT (Cao et al., 2016), oxidative stress (Giannoni et al., 2008), and adjusting their integrin levels (Hynes, 1992). Lots of research demonstrates that anoikis is essential in the development of a multiple cancers, like renal cancer (Wang et al., 2022), lung cancer (Jin et al., 2018), and gastric cancer (Ye et al., 2020) as well as PCa (Nepali and Kyprianou, 2023). Prior studies reported that several anoikis-related genes (ARGs) were closely correlated to the metastasis and invasion of PCa (Rennebeck et al., 2005; Lee et al., 2021). For example, FLIP, an inhibitor of anoikis, was found upregulated in a mouse model of PCa metastasis (Mawji et al., 2007). Inhibiting the synthesis of FLIP protein reduces the formation of distant tumors (Mawji et al., 2007). Moreover, focal adhesion complex protein Talin1 was a prognostic biomarker, which promote the migration and invasion of PCa through focal adhesion signaling and resistance to anoikis (Sakamoto et al., 2010). Furthermore, the expression of CENPF gene was upregulated in human PC3 cells, and silencing CENPF increases sensitivity to anoikis-induced apoptosis (Shahid et al., 2018). In addition, AR may suppress cell deaths via anoikis and entosis, potentially leading increased PCa metastasis (Wen et al., 2014). There is still a necessity to identify new genetic markers related to anoikis, which could serve as a foundation for risk stratification in patients with BCR of PCa.

**FIGURE 1:**
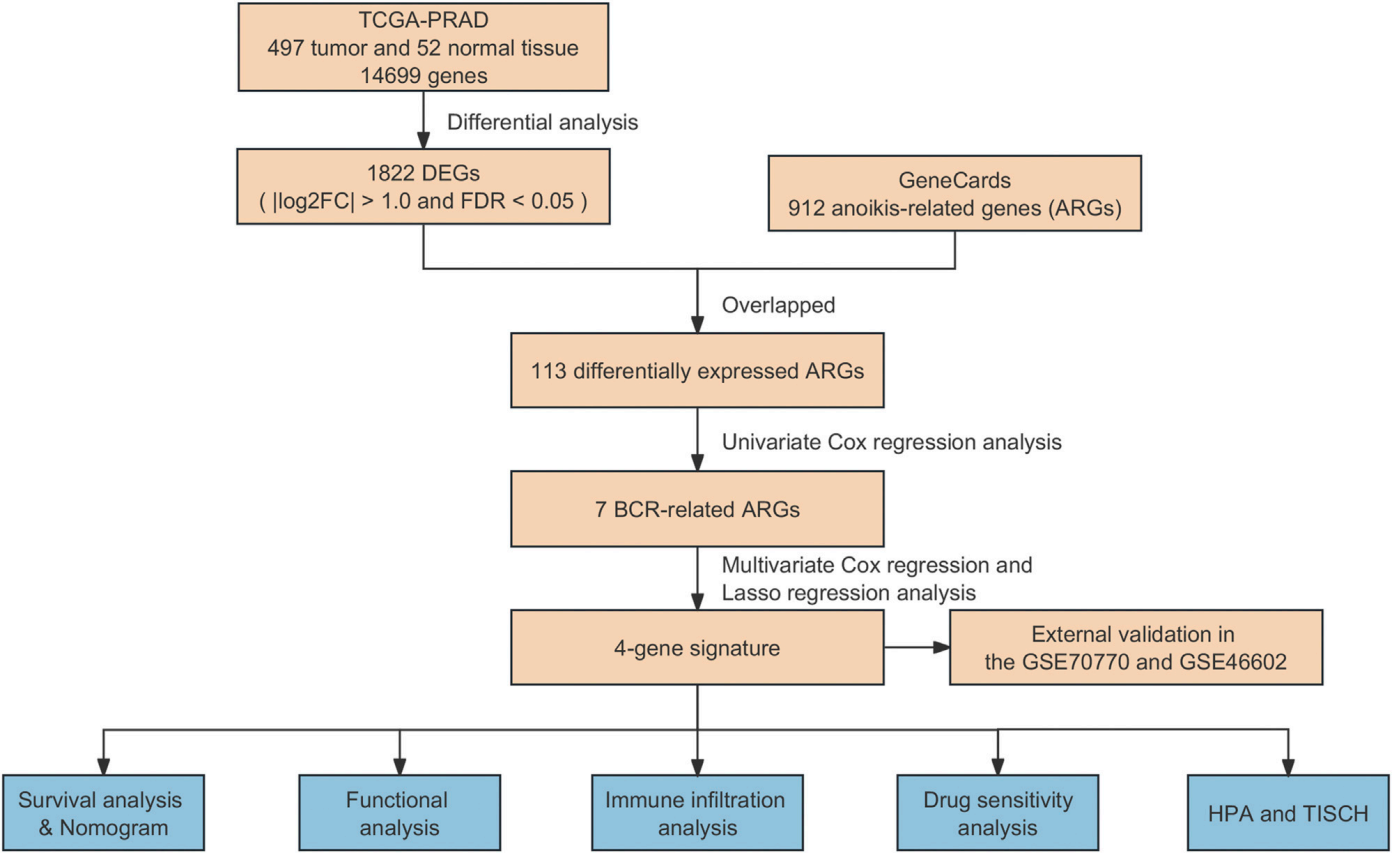
Flowchart of data collection and study design.

In this study, we tried to explore the association between ARGs and the risk of biochemical recurrence in PCa. For this purpose, we employed differentially expressed ARGs from TCGA and the GeneCards databases, establishing an ARG-based signature to forecast biochemical recurrence outcomes in PCa. Moreover, the predictive capacity of ARGs in assessing patient prognosis using Gene Expression Omnibus (GEO) datasets (GSE70770 and GSE46602) were validated. Furthermore, the molecular and immune characteristics, sensitivity to antineoplastic agents, and the effectiveness of immunotherapy associated with the model in PCa were assessed. Finally, the protein expression of prognostic genes was confirmed based on the Tumor Immune Single-Cell Hub (TISCH) and the Human Protein Atlas (HPA). The workflow of this research was illustrated in Figure 1.

## 2 Materials and methods

### 2.1 Data collection

The clinical information and transcriptome matrix, encompassing 497 prostate tumor samples and 52 adjacent-normal samples, were acquired from TCGA database (https://portal.gdc.cancer.g-ov/). The expressed genes (DEGs) were differentially detected using the “limma” R package, employing thresholds of |log2FC| > 1.0 and FDR <0.05. Furthermore, we obtained two external validation cohorts, GSE70770 and GSE46602, from the Gene Expression Omnibus (GEO) datasets available at https://www.ncbi.nlm.nih.gov/geo/. In addition, a sum of 912 genes related to anoikis (ANOIKIS-related genes or ARGs) was procured from the GeneCards database (https://www.genecards.org/). The ARGs that were differentially expressed were then retrieved by overlapping with the DEGs.

### 2.2 Construction of risk score model

In this study, 429 PCa patients were randomly allocated into training and testing sets at a 1:1 ratio, and the training set was employed to develop a risk score model based on ARGs. Univariate Cox analysis, employing the R package “survival”, was conducted to identify ARGs significantly associated with BCR of PCa at a significance criterion of *p* < 0.05. Subsequently, to mitigate the risk of overfitting the model, the least absolute shrinkage and selection operator (LASSO) regression algorithm along with 10-fold cross-validation were employed to narrow down the candidate ARGs. Finally, multivariate Cox analysis was performed to select ARGs independently predicting the prognosis of PCa. A risk model was then constructed based on the prognostic ARGs, with the formula: (coefficient × the expression of EEF1A2) + (coefficient × the expression of RET) + (coefficient × the expression of FOSL1) + (coefficient × the expression of PCA3).

Additionally, to stratify all qualified individuals into high- and low-risk groups, the median risk score was used as the threshold. To illustrate the survival discrepancies between individuals in high- and low-risk categories, Kaplan-Meier survival analysis was conducted, utilizing the “survival” and “survminer” packages in R. The model’s performance to predict was assessed by conducting the time-dependent receiver operating characteristic (ROC) curve analysis, executed with the “timeROC” package in R. Validation of the model was also carried out in the independent validation set.

### 2.3 Validation of risk model

To confirm the effectiveness of the prognostic model based on ARGs, the GSE70770 and GSE46602 cohorts were selected as external validation sets. Using the pre-established formula, the risk score of each sample in these two cohorts was calculated. Subsequently, based on the median risk score, all samples from the two datasets were grouped as either low- or high-risk group.

### 2.4 Construction of ARGs-based nomogram

The clinical and pathological features, encompassing aspects like age, T stage, N stage, and risk score, were compiled and utilized to develop a nomogram model through the use of the “rms” package in R. This model aimed to estimate the possibility of BCR-free survival in PCa patients at 3, 5, and 8 years. Furthermore, univariate and multivariate Cox regression analyses were employed to identify independent prognostic factors from the risk score and various Clinicopathological features.

### 2.5 Functional enrichment analysis

To analyze the differentially expressed genes (DEGs), “clusterProfiler” R package and the “ggplot2” R package were used to identified the biological processes and signaling pathways based on Gene Ontology (GO), and Kyoto Encyclopedia of Genes and Genomes (KEGG) dataset. Additionally, the “c2.cp.reactome.v7.4.symbols.gmt” was obtained from the MSigDE database, and the reactome pathway-based analysis was carried out using the “GSVA” R package and the “pheatmap” R package.

### 2.6 Analysis of immune infiltration landscape

The CIBERSORT algorithm utilizes the principle of linear support vector regression to deconvolve the expression matrix of immune cell subtypes to estimate the abundance of immune cells (Newman et al., 2015). To examine the prevalence of diverse immune cell types in the low- and high-risk groups, the CIBERSORT and CIBERSORTx (https://cibersortx.stanford.edu/) were applied to estimate the level and proportion of infiltration of different immune cells. Additionally, a single-sample gene set enrichment analysis (ssGSEA) algorithm was also used to evaluate the differences in immune cell infiltration levels between the different risk groups, utilizing the “GSVA” package in R. The disparities between the low-risk and high-risk groups were visualized using the “ggplot2” package in R. In addition, the correlation between the risk score values with the presence of immune infiltrating cells was also investigated using CIBERSORT method.

### 2.7 Immunotherapy response and drug sensitivity analysis

The immune evasion potential in PCa patient samples was evaluated using the Tumor Immune Dysfunction and Exclusion (TIDE) algorithm based on data from the TIDE database (http://tide.dfci.harvard.edu/login/). The half maximal inhibitory concentration (IC50) and the expected response of PCa patients to anticancer drugs were calculated with the “oncoPredict” package in R utilizing data from the Genomics of Drug Sensitivity in Cancer (GDSC) database. In addition, the outcomes were visually represented with the “ggplot2” package in the R programming language.

### 2.8 Validation of prognostic genes

The Tumor Immune Single-Cell Hub (TISCH; http://tisch.comp-genomics.org) was utilized for a comprehensive investigation of the tumor microenvironment (TME) heterogeneity, encompassing a wide range of datasets and cell types. Therefore, we exploited the single-cell RNA sequencing dataset PRAD_GSE141445 (Chen et al., 2021) from the TISCH database for the analysis of the expression of identified ARGs in the TME. To confirm the protein expression of prognostic genes specifically in PCa tissues, immunohistochemistry data were cross-referenced with the Human Protein Atlas (HPA; https://www.proteinatlas.org/).

### 2.9 Statistical analysis

Continuous variables were evaluated using either the Wilcoxon test or the t-test, depending on their suitability for the data. Spearman correlation analysis was employed for correlational analyses. Kaplan-Meier survival analysis was utilized for the generation of survival curves, and comparisons between these curves were conducted employing the log-rank test. A significance level of *p* < 0.05 was considered suitable for all statistical assessments. All statistical analysis were performed using R software (version 4.3.0).

## 3 Results

### 3.1 Identification of differentially expressed ARGs

**FIGURE 2:**
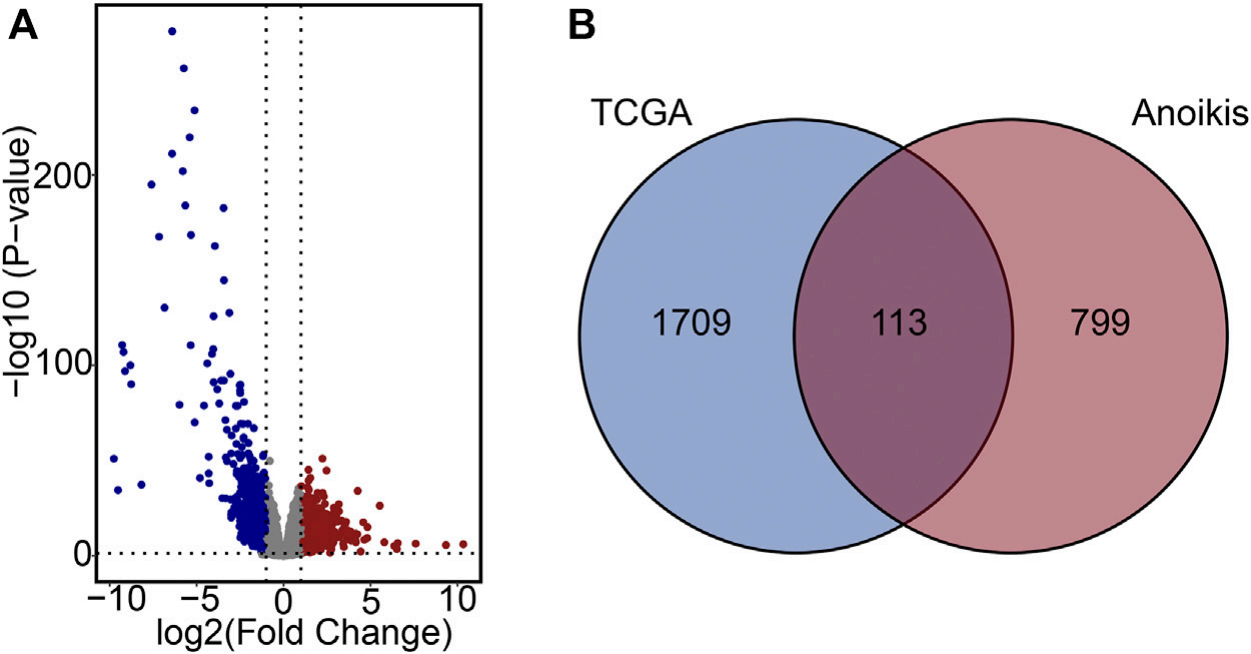
Characterization of differentially expressed ARGs. **(A)** Volcano plot of DEGs in PCa. **(B)** Venn diagram of DEGs and ARGs.

Gene expression data from both PCa samples and normal tissues in the TCGA-PCa database were subjected to analysis, with 1822 DEGs identified, comprising 1,052 downregulated and 770 upregulated genes (Figure 2A). Subsequently, these DEGs overlapped with the 912 ARGs extracted from GeneCards. Finally, a total of 113 differentially expressed ARGs was applied for further analysis. (Figure 2B).

### 3.2 Risk model construction based on the ARGs prognostic signatures

**FIGURE 3:**
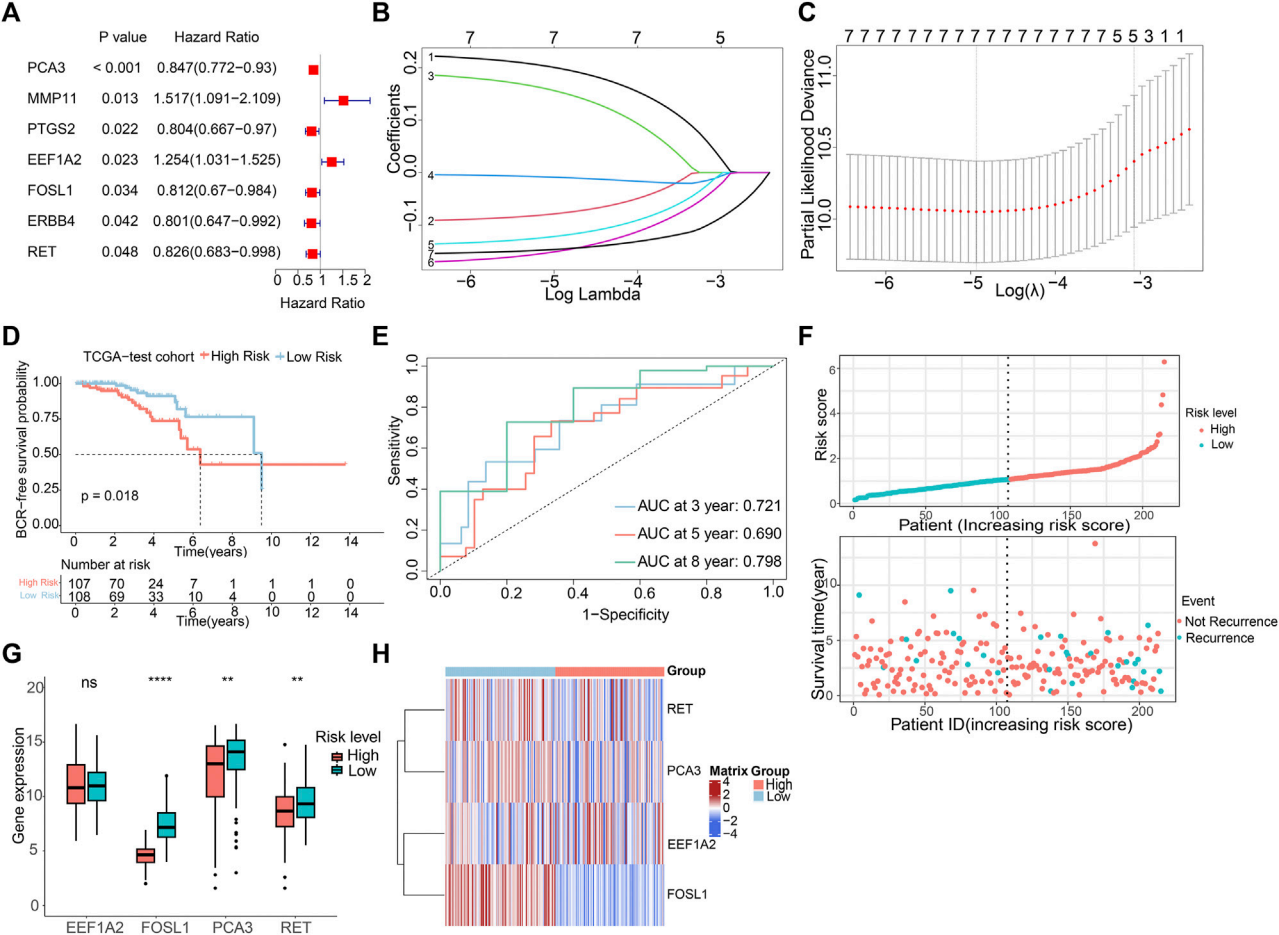
Construction of risk model risk model based on the prognostic ARGs in PCa. **(A)** Univariate Cox regression analysis of the ARGs. **(B,C)** LASSO (least absolute shrinkage and selection operator) regression. **(D)** K-M curves of BCR outcome between high- and low-risk score groups in the testing set. **(E)** ROC curves for BCR-free predictive performance at 3-, 5-, and 8-year in the testing set. **(F)** The risk score distribution and BCR status of PCa patients in the testing. **(G,H)** The boxplot and risk heatmap of the prognostic ARGs in the testing set.

To create a predictive signature for the biochemical recurrence status in PCa patients, 429 individuals were randomly divided in equal proportions into a training group (comprising 214 patients) and a testing set (consisting of 215 patients). As shown in Figures 3A–C, univariate Cox regression analysis and LASSO analysis were conducted in the training set, identifying 7 ARGs associated with the BCR rate. Moreover, the multivariate Cox regression analysis found that 4 ARGs independently predictive of PCa prognosis, which were selected to develop a risk prediction model (Supplementary Figure S1A). The risk score was computed utilizing the following formula: (0.261×EEF1A2 expression) + (−0.142×RET expression) + (−0.196×FOSL1 expression) + (−0.184×PCA3 expression). Following this, individuals with PCa were stratified according to the median risk score, and then categorized into high- and low-risk groups. In the training set, analysis of Kaplan-Meier survival curves implied that individuals with elevated risk scores experienced a notably reduced rate of BCR-free survival (Supplementary Figure S1B). The AUC were 0.709, 0.765, and 0.808 for 3-, 5-, and 8-year BCR-free rates, respectively (Supplementary Figure S1C). Risk plots revealed a favorable relation between rising risk scores and the prevalence of BCR in PCa (Supplementary Figure S1D). Furthermore, the expression profiles of the four prognostic ARGs in both the high-risk and low-risk groups were graphically depicted using box plots and a risk heatmap. These findings revealed that individuals with an elevated risk score demonstrated increased expression of EEF1A2. In contrast, PCA3, RET, and FOSL1 showed higher expression levels in the low-risk group, as illustrated in Supplementary Figure S1E. The testing set was then utilized for verification of the predictive accuracy of the risk model. In Figures 3D,E, it is shown that there are significant differences in the BCR-free survival rates among different risk groups in the testing set. The AUC values for the BCR-free survival rates at 3, 5, and 8 years are 0.721, 0.690, and 0.798, respectively, indicating that the risk model based on ARGs has a good predictive performance. Figures 3F–H display the risk distribution plots, gene expression box plots, and risk heatmap of different risk groups in the testing set.

### 3.3 Independent prognosis analysis of the ARGs prognostic signature

**FIGURE 4:**
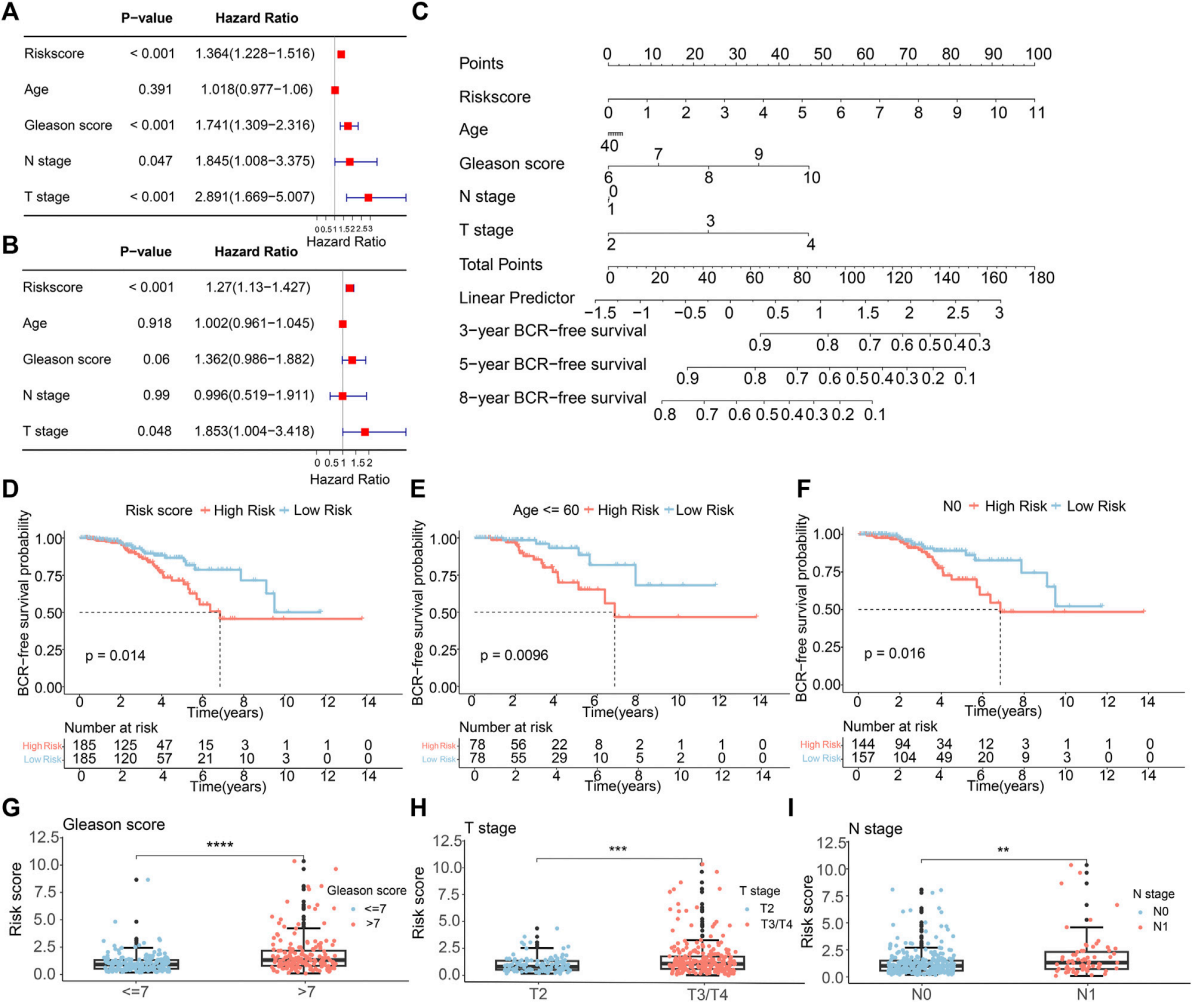
The independence of the ARGs prognostic signatures for PCa. **(A)** Univariate Cox analysis and **(B)** multivariate Cox analysis shows the correlation of the BCR-free occurrence and risk score, and clinicopathological factors. **(C)** A nomogram based on the ARGs and clinicopathological factors. **(D)** The K-M survival analysis in the different risk groups of PCa patients in the entire TCGA sets. The BCR-free rate of patients with PCa in the low- and high-risk group among the **(E)** Age≤60; **(F)** N0. The box plots of correlation between risk scores and clinicopathological characteristics, including **(G)** Gleason score; **(H)** T stage; **(I)** N stage.

We conducted both multivariate and univariate Cox regression analyses on the comprehensive TCGA-PCa dataset to evaluate the independence of the ARGs prognostic signatures for PCa. The univariate Cox regression analysis unveiled a notable connection between the BCR rate in PCa and factors such as risk score (HR = 1.364, *p* < 0.001), Gleason score (HR = 1.741, *p* < 0.001), N stage (HR = 1.845, *p* = 0.047), and T stage (HR = 2.891, *p* < 0.001) (Figure 4A). The multivariate Cox regression analysis showed that the risk score (HR = 1.27, *p* < 0.001) and T stage (HR = 1.853, *p* = 0.048) were independent prognostic indicators for PCa (Figure 4B). Subsequently, we developed a new nomogram based on clinical pathological features and risk score to predict the 3-, 5-, and 8-year BCR-free survival probabilities of PCa patients (Figure 4C). To explore the prognostic value of the risk score based on ARGs in different clinicopathological factors, we conducted stratified subgroup analyses. As illustrated in Figure 4D, risk stratification of the entire TCGA cohort showed that patients in the high-risk group had notably poorer outcomes. Moreover, PCa patients with different clinical pathological characteristics were divided into high-risk and low-risk groups based on the prognostic signature of ARGs. Kaplan-Meier analysis displayed that the occurrence of BCR showed a notably higher level in individuals with a high-risk score in contrast to those in the low-risk group, particularly among individuals aged ≤60 years and those with N0 status (Figures 4E,F). However, due to small sample size or uneven distribution, in patients aged >60, with different Gleason scores, and varying N and T stages, there was no significant difference in BCR incidence (Supplementary Figure S2). Furthermore, Figures 4G–I illustrated that higher risk scores are indeed positively correlated with various adverse clinicopathological characteristics, including higher Gleason scores, higher T stages, and lymph node metastasis. These results indicated that the risk score derived from ARGs can act as a prognostic factor that acts independently in individuals with PCa. It could be effectively used to predict the likelihood of BCR-free survival in PCa patients.

### 3.4 Validation of risk model based on GEO cohorts

**FIGURE 5:**
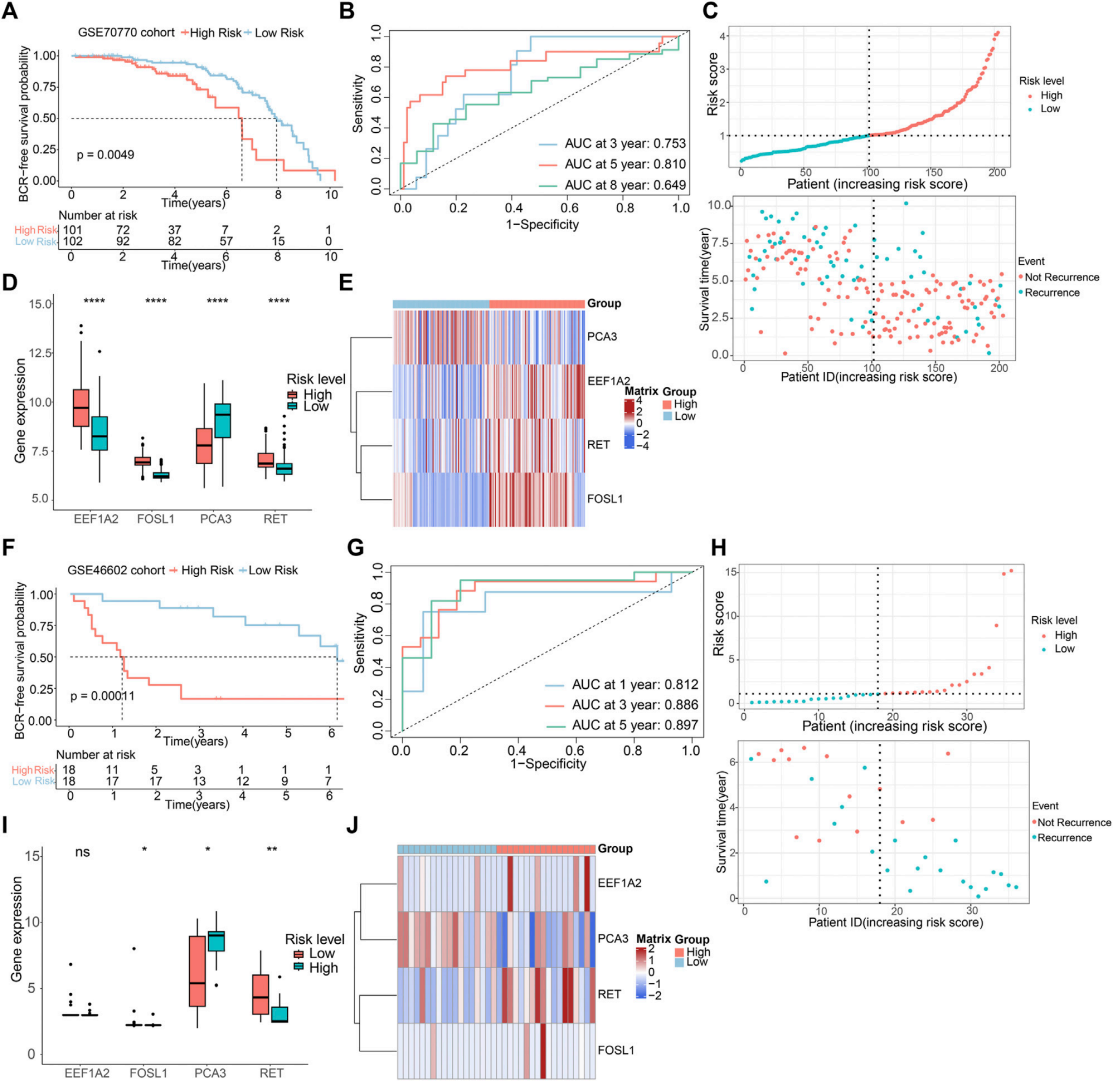
Validation of risk model based on GEO cohorts. K-M curves of BCR-free rate between high- and low-risk score groups in the **(A)** GSE70770, **(F)** GSE46602. The AUC of the risk model in the **(B)** GSE70770, **(G)** GSE46602. The risk score distribution and BCR status of PCa patients in the **(C)** GSE70770, **(H)** GSE46602. The boxplots of the prognostic ARGs in the **(D)** GSE70770, **(I)** GSE46602. The risk heatmap of the prognostic ARGs in the **(E)** GSE70770, **(J)** GSE46602.

To assess the predictive precision of the risk model, we applied the same methodologies to the GSE70770 and GSE46602 cohorts, utilizing them as external validation sets. Multivariate Cox regression analysis in both cohorts exhibited the independence of the ARGs prognostic signatures (Supplementary Figure S3). Survival analysis unveiled that patient with low-risk scores displayed notably higher rates of BCR-free survival in comparison to those with high-risk scores (Figures 5A,F), aligning with the observations from the TCGA dataset. The AUC values in the GSE70770 dataset were 0.753, 0.810, and 0.649 for 3-, 5-, and 8-year BCR-free survival predictions, respectively. The AUC values for 1-, 3-, and 5-year BCR-free occurrence in the GSE46602 dataset were 0.812, 0.886, and 0.897, respectively (Figures 5B,G). Figures 5C–E,H–J visually represents the distribution of risk scores, the expression levels of the four prognostic ARGs, and the BCR status of individuals within the two external validation sets. These data implied that the risk model performs well in terms of prognosis.

### 3.5 Functional enrichment analysis of the differentially expressed ARGs

**FIGURE 6:**
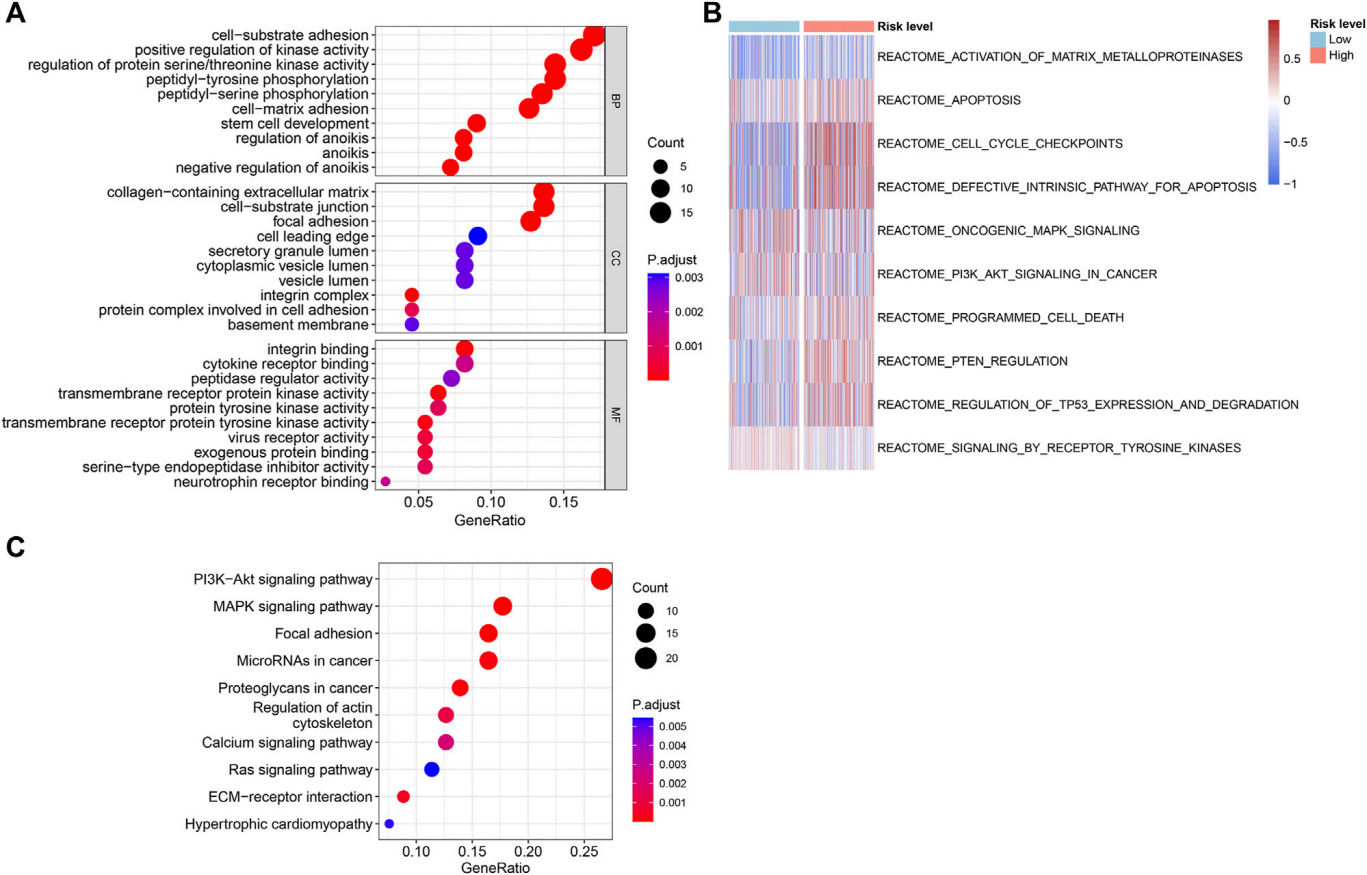
Functional enrichment analysis of the differentially expressed ARGs in the different risk groups. **(A)** The results of GO enrichment analysis. **(B)** GSVA analysis shows the REACTOME term of PCa patients in the different risk groups. **(C)** KEGG enrichment analysis of the differentially expressed ARGs.

The underlying biological functions and molecular mechanisms of ARGs were revealed using functional enrichment analysis. ARGs in PCa were shown to be significantly abundant in processes such as cell-substrate adhesion, peptidyl-tyrosine phosphorylation, control of protein serine/threonine kinase activity, cell-matrix adhesion, and anoikis regulation (Figure 6A). The findings of the GSVA enrichment analysis revealed the signature signaling pathways of ARGs for PCa patient in the high- and low-risk groups (Figure 6B). According to KEGG analysis, ARGs in PCa were largely implicated in the PI3K-Akt signaling pathway, the MAPK signaling pathway, and focal adhesion (Figure 6C). The outcomes implied that ARGs might have a substantial role in the migration and metastasis of PCa.

### 3.6 Immune infiltration analysis

**FIGURE 7:**
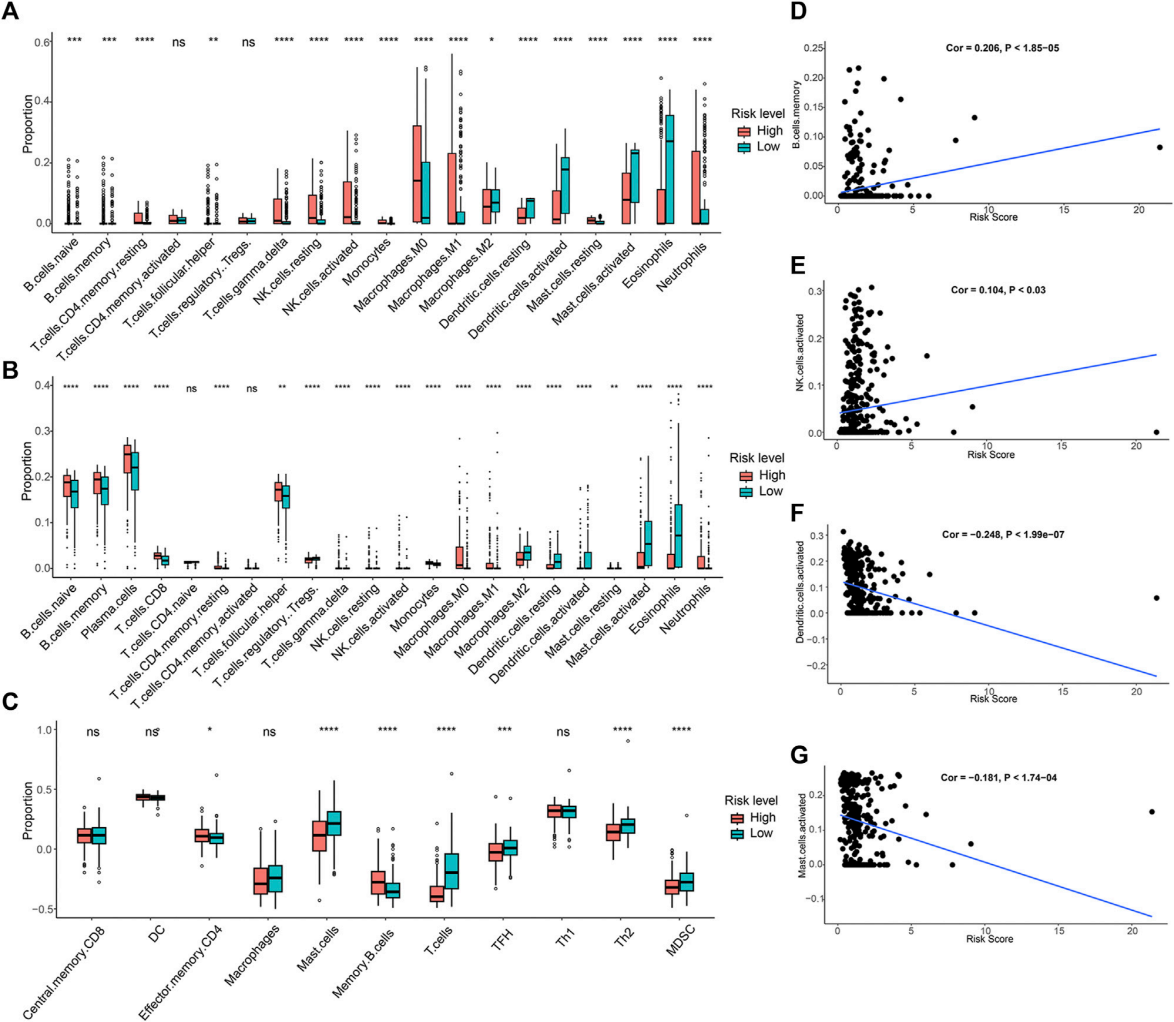
Immune infiltration analysis of PCa patients in risk groups. **(A)** The fraction of 19-type immune cells in different risk groups. **(B)** Differences in immune infiltration of 22-type immune cells. **(C)** The proportion of 11-type immune cells in different risk groups. **(D–G)** The correlations between the four immune cells abundance and risk scores.

For assessing the pattern of immune cell infiltration in patients with PCa, the CIBERSORT, CIBERSORTx and ssGSEA algorithms were applied, focusing on two risk subgroups. The CIBERSORT analysis showed that the high-risk group displayed a considerably greater proportion of most immune cells. In contrast, the low-risk group had a larger percentage of resting dendritic cells, activated dendritic cells, activated mast cells, and eosinophils, as depicted in Figure 7A. CIBERSORTx is an updated version of CIBERSORT, and the results of both analyses were relatively consistent. Notably, CIBERSORTx identified more cell types, such as plasma cells and CD8 T cells, which showed higher infiltration levels in the high-risk group (Figure 7B). The ssGSEA algorithm demonstrated that individuals with a low-risk score showed a larger portion of mast cells, T cells, T follicular helper cells (TFH), T helper two cells (Th2), and myeloid-derived suppressor cells (MDSC). Conversely, the fraction of effector memory CD4 cells and memory B cells was greater in the high-risk group (Figure 7C). A correlation analysis was conducted to investigate the relationship between the abundance of immune cells and the risk score. This analysis revealed that the risk score was in a positive relationship with the presence of memory B cells and activated NK cells, while it showed a negative association with activated dendritic cells and mast cells (Figures 7D–G). Overall, our data suggested a correlation between ARGs risk model and the immune infiltration landscape in patients with PCa.

### 3.7 Immunotherapy response analysis

**FIGURE 8:**
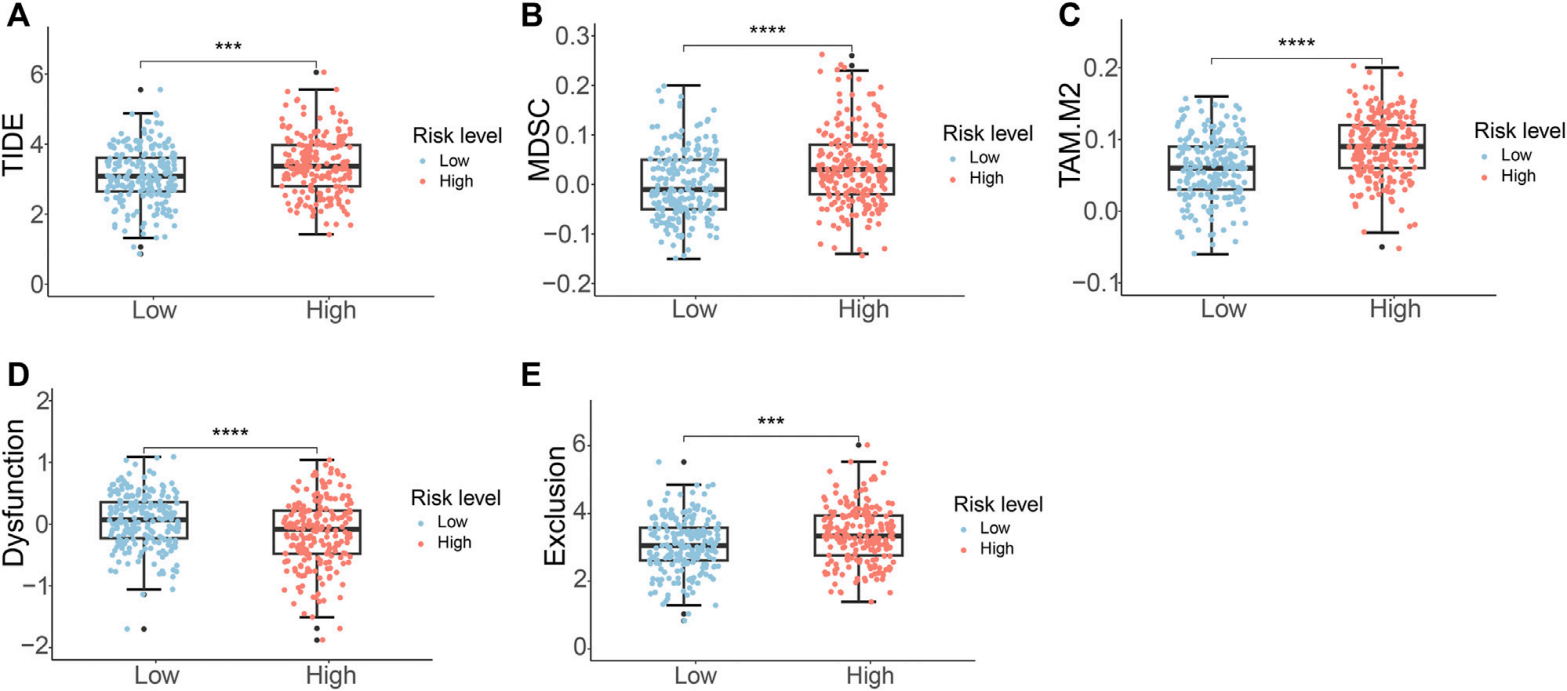
Immunotherapy response analysis of PCa patients. **(A)** TIDE score **(B)** MDSC score **(C)** TAM.M2 score **(D)** T-cell dysfunction score **(E)** T-cell exclusion score.

Given the wide range of immune infiltration among PCa patients, we investigated the response to immunotherapy in distinct risk groups. The TIDE analysis indicated that high-risk PCa patients exhibited notably higher TIDE scores in contrast to individuals in the low-risk group (Figure 8A). This suggested that individuals with a high-risk score might have a diminished reaction to immune checkpoint therapy. Additionally, the high-risk group demonstrated elevated scores for MDSC, TAM.M2, and exclusion, while showing a lower dysfunction score (Figures 8B–E).

### 3.8 Drug sensitivity analysis

**FIGURE 9:**
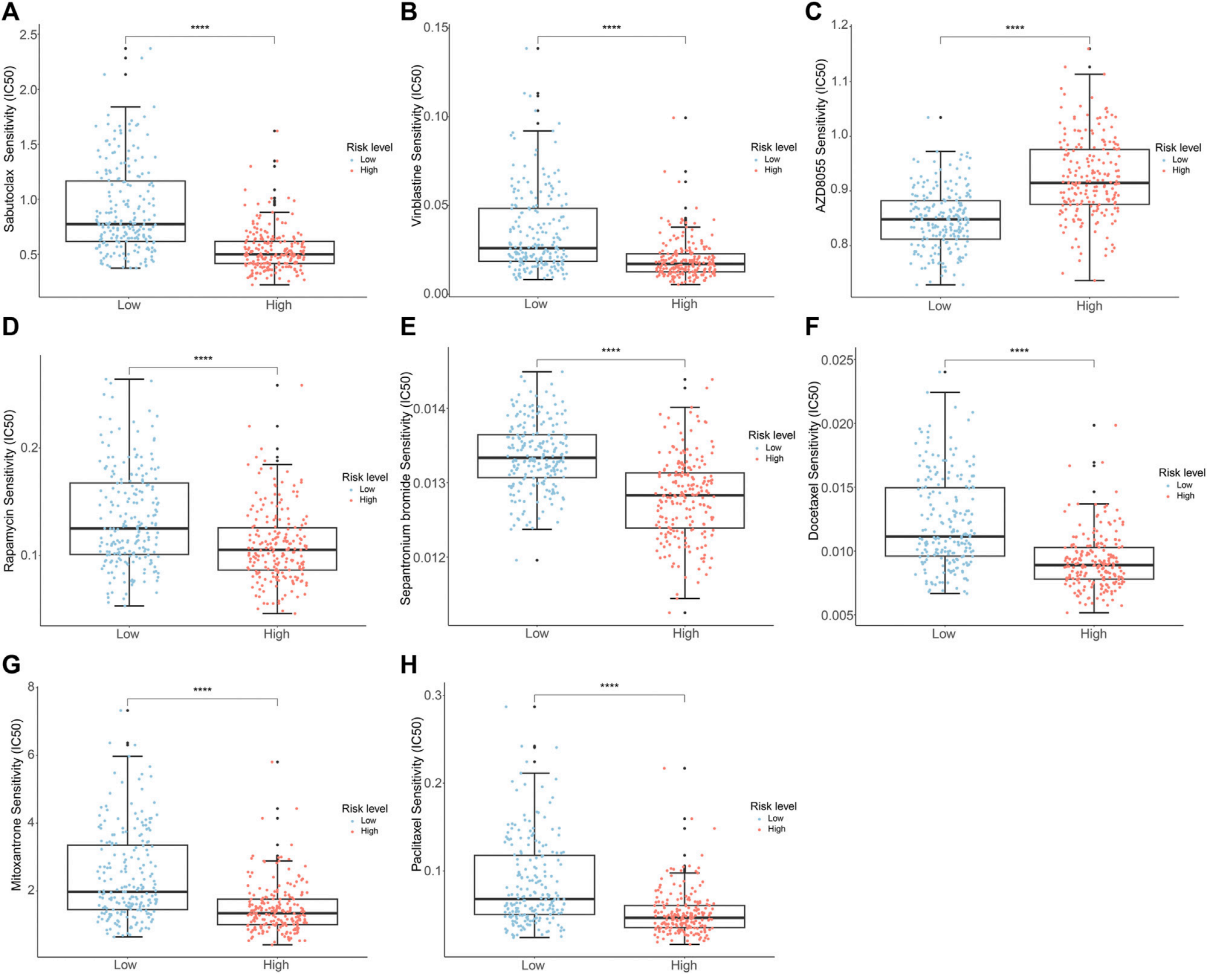
Analysis of drug sensitivity of PCa patients in the different risk groups. **(A)** Sabutoclax **(B)** Vinblastine **(C)** AZD8055 **(D)** Rapamycin **(E)** Sepantronium Bromide (YM155) **(F)** Docetaxel **(G)** Mitoxantrone **(H)** Paclitaxel.

To evaluate the response to chemotherapeutic drugs in the two subgroups, we carried out a drug sensitivity analysis. The outcomes indicated that the IC50 values of sabutoclax, vinblastine, rapamycin, sepantronium bromide (YM155), docetaxel, mitoxantrone, and paclitaxel were notably greater in the low-risk group. However, it was observed that the high-risk group had a higher IC50 for AZD8055, as indicated in Figures 9A–H. The findings highlighted the diverse efficacy benefits of the selected chemotherapeutic drugs on individuals with PCa in various risk groups and could offer guidance for the selection of chemotherapy for PCa patients.

### 3.9 Validation of prognostic genes

**FIGURE 10:**
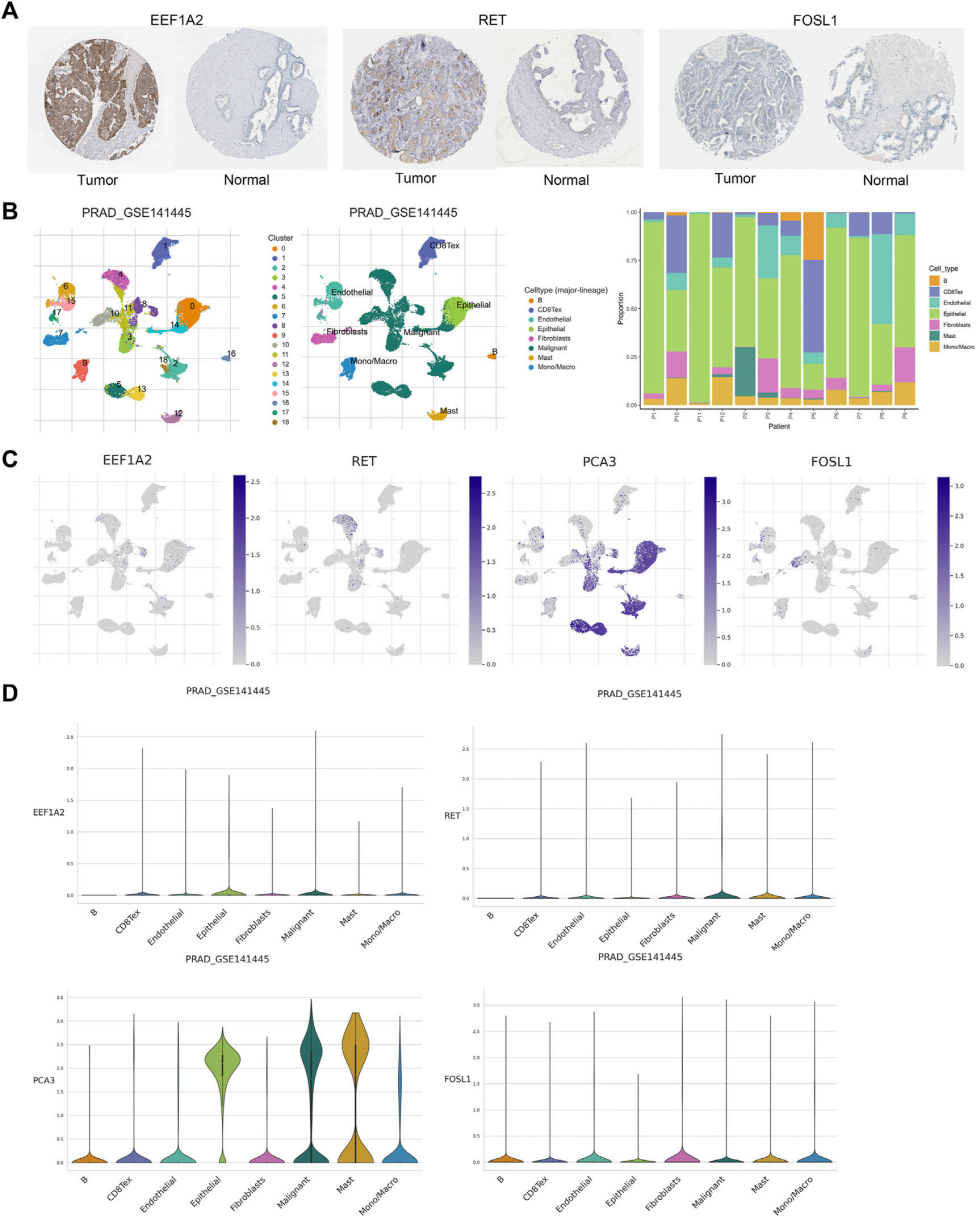
Validation of prognostic genes of ARGs. **(A)** Based on the HPA database, the immunohistochemical results for EEF1A2, RET and FOSL1. **(B)** Annotations for all cell types in GSE141445 and the proportion of each cell type. **(C, D)** Proportions and expressions of FOSL1, EEF1A2, PCA3 and RET.

Based on the information available on the HPA website, we observed that the EEF1A2 gene in the risk model exhibited high expression in PCa cases, while the RET and FOSL1 genes showed moderate or weak expression (Figure 10A). Furthermore, we leveraged the single-cell RNA sequencing dataset GSE141445 from the TISCH database to investigate the expression of four ARGs within the TME. The GSE141445 dataset comprise 18 cell clusters and eight intermediate cell types, the distribution and quantity of these clusters were depicted (Figure 10B). Figures 10C,D showed the expression levels of the genes EEF1A2, RET, FOSL1, and PCA3 in each cell type within the dataset. These data indicated that these four ARGs may contribute in the tumor microenvironment of PCa.

## 4 Discussion

Early recurrence is associated with a heightened risk of metastasis in PCa, with 24%–34% of patients with BCR progressing to metastatic disease (Pound et al., 1999; Boorjian et al., 2011). Anoikis pertains to a distinctive form of programmed cell death that is initiated when cells disconnect from the extracellular matrix and become isolated from neighboring cells. This mechanism significantly contributes to inhibiting tumor invasion and metastasis. Consequently, evaluating BCR risk based on the anoikis signature is essential for improving the accuracy of PCa prognosis.

In this research, we identified four ARGs that are associated with the risk of BCR in PCa. Utilizing these genes, we constructed a risk model to forecast BCR in PCa using the TCGA dataset, and subsequently, we verified the model in two GEO datasets. Kaplan-Meier survival analysis exhibited a significantly higher risk of BCR in the high-risk group in comparison to the low-risk group. ROC analysis confirmed the effectiveness of the prognostic model in predicting BCR events at 3-, 5-, and 8-year intervals. It was worth noting that the majority of BCR times for patient samples in the GSE46602 dataset were less than 8 years. Consequently, we conducted ROC analysis to validate the prognostic model’s performance in predicting 1-, 3-, and 5-year BCR events. The multivariate and univariate Cox regression analyses showed that the risk score derived from ARGs could serve as a standalone prognostic indicator for forecasting BCR-free survival in individuals with PCa. Additionally, we carried out a nomogram to visually represent the influence of the risk score and various clinicopathological characteristics on 3-, 5-, and 8-year BCR-free survival. Taken together, the risk model, based on 4 ARGs, demonstrated accurate assessment capabilities for predicting the BCR risk in PCa patients.

In this study, we identified 4 ARGs for the construction of a BCR risk model in PCa. Among these ARGs, patients with a high-risk score reported greater EEF1A2, RET, and FOSL1 levels, but PCA3 expression was reduced. Previous investigations elucidated some correlations between these genes and the tumorigenesis and pathophysiology of cancer. EEF1A2, a coding gene crucial for protein translation elongation, has been demonstrated to exhibit altered expression in numerous cancers (Cristiano, 2022), which actively participate in the initiation and progression of various cancer types during carcinogenesis (Anand et al., 2002; Hassan et al., 2020; Jia et al., 2021). EEF1A2 was reported to facilitate the migration, invasion, and metastasis of pancreatic cancer cells by activating Akt and upregulating MMP-9 expression (Xu et al., 2013). Inhibiting EEF1A2 leaded to a significant upregulation of apoptotic pathway proteins (caspase3, BAD, BAX, PUMA), while elevated levels of EEF1A2 promoted the proliferation and suppress apoptosis of PCa cells (Sun et al., 2014). Worst et al. observed that EEF1A2 was overexpressed in PCa with higher Gleason scores, and patients with increased EEF1A2 expression had markedly shortened BCR-free survival (Worst et al., 2017). These findings implied that EEF1A2 played a role in the transformation and progression of PCa, which was consistent with our observations of elevated EEF1A2 expression in high-risk populations. RET is a receptor tyrosine kinase, which can mediate cell proliferation, survival, migration, and is associated with the progression of various tumors (Hu et al., 2023). Our data indicated that high expression of RET was linked to poor prognosis in PCa patients. It was reported that RET expression played a crucial role in the survival, proliferation, and anoikis resistance of medullary thyroid carcinoma cells (Lian et al., 2017). In PCa, RET is expressed in all PCa cell lines, and RET signaling can activate the AKT or ERK pathways, promoting PCa transformation-related phenotypes by activating the p70S6 kinase (Ban et al., 2017). Knocking down or pharmacologically inhibiting the RET kinase in various mouse and human neuroendocrine PCa models significantly diminished PCa tumor growth and cellular vitality (VanDeusen et al., 2020). These results suggested that RET was conducive to the development of malignant characteristics in PCa cells, and further investigation of the clinical potential of RET gene was warranted. The FOSL1 gene is responsible for encoding Fos-related antigen 1 (FRA1), which is elevated in breast cancer, colorectal cancer, lung cancer, and various other malignancies (Jiang et al., 2020). Our data also indicated FOSL1 gene was biomarker high expressed in PCa patients. It was reported that the oncogene K-Ras elevated the expression of ITGA6 through FOSL1 inducing resistance to anoikis (Zhang et al., 2017). High expression of FOSL1 in PCa could enhance the proliferation and metastasis of PCa cells by modulating the EMT pathway (Luo et al., 2018). Therefore, as a promoter of EMT, FOSL1 may have a significant impact on PCa carcinogenesis. Prostate Cancer Antigen 3 (PCA3) is a specific type of long non-coding RNA (lncRNA) found in the prostate. The levels of PCA3 in urine were commonly used as diagnostic biomarkers for PCa (Lemos et al., 2019). Lauer et al. found that the upregulation of lncRNA PCA3 and the downregulation of PRUNE2 might be early (rather than late) molecular events in the progression of prostate tumors, but were unrelated to BCR (Lauer et al., 2023). Another study also suggested that PCA3 appeared to be an important marker for early-stage or less invasive tumors of PCa, however, the gene expression levels of PCA3 and PRUNE2, as well as the PCA3/PRUNE2 ratio, could differentiate whether patients experience BCR events (Dias-Neto et al., 2017). The differences in BCR outcomes between these two studies might be attributed to the cohorts included. Our findings were in line with the latter results, indicating that PCA3 expression was higher in the group of individuals with low risk of BCR compared to that in the high-risk group. In summary, EEF1A2, RET, and FOSL1 could serve as prognostic risk factors for PCa. Conversely, PCA3 was negatively correlated with PCa prognosis. The risk model based on these four ARGs not only could be a prognostic marker for BCR in PCa but also had the potential to be a novel prospective target gene for PCa treatment.

We also conducted GO, KEGG, and GSEA enrichment studies on the ARGs to better understand their putative biological roles and molecular processes. As anticipated, these genes exhibited close associations with the biological processes involving the regulation of anoikis and were also linked to the signaling pathways of PI3K-Akt and MAPK. According to current research reports, silencing eEF1A2 significantly reduced the occurrence of hepatocellular carcinoma by inhibiting the PI3K/Akt/NF-κB signaling transduction (Qiu et al., 2016). Activating RET alterations have been shown to enhance the activity of the PI3K/AKT and MAPK pathways, thereby promoting the proliferation and development of various types of tumor cells (Brzezianska and Pastuszak-Lewandoska, 2011; Regua et al., 2022). In melanoma, sustained expression of FOSL1 was associated with continuous overactivation of the MAPK pathway (Maurus et al., 2017). Further, the process of anoikis resistance reported in several studies is indeed related to the pathways we identified. Anoikis resistance has been reported to induce alterations in the Ras/ERK and PI3K/Akt signaling pathways, as well as matrix remodeling, in endothelial cells (de Sousa Mesquita et al., 2017). Depleting mitochondrial DNA in prostate epithelial cells induces the cell to develop anoikis resistance and enhances its invasive ability by activating the PI3K/Akt2 signalling pathway (Moro et al., 2009). Activation of the Src kinase-mediated MAPK pathway was associated with angiogenesis in osteosarcoma cells anoikis resistance (Gao et al., 2019). These studies suggested that ARGs may play a role in the anoikis process of PCa by regulating the PI3K-Akt and MAPK signaling pathways, further investigation is necessary to validate this hypothesis.

Infiltrating immune cells serves a complex biological function in PCa development. Studies have shown that low levels of mast cells in cancer tissue are associated with poor outcomes such as BCR and metastasis in PCa (Hempel et al., 2017; Sfanos, 2022). This is consistent with our results of less mast cell infiltration in the high-risk group. Our study found that the BCR high-risk group had a higher proportion of M1 macrophages, which is consistent with previous research, indicating that infiltrating M1 macrophages are an important adverse prognostic factor for BCR in PCa (Andersen et al., 2021). Moreover, this study also showed that the infiltration level of neutrophils significantly increases in the high-risk group. A study has shown that enhancing the cytotoxicity of neutrophils in the bone might be beneficial for the treatment of patients with bone metastatic PCa (Costanzo-Garvey et al., 2020). Therefore, neutrophils may have important therapeutic implications for bone metastasis patients in the high-risk group. Furthermore, previous studies have reported high levels of tumor-infiltrating CD8 T cells associated with BCR in PCa (Ness et al., 2014), as well as specimens from patients in the high-risk group and those with recurrence or progression of PCa showing more B-cell infiltration (Woo et al., 2014). These findings are consistent with our results, indicating a higher level of infiltration of CD8 T cells and B cells in the high-risk group for BCR. It is worth noting that although the samples in the single-cell RNA sequencing dataset were not risk stratified, the proportions of B cells and CD8^+^ T cells in them were similar to those in the high-risk group in immune infiltration, and these immune cells may play a similar role in TME of PCa.

TIDE analysis was used to evaluate the potential clinical efficacy of immunotherapy in different risk groups. An elevated TIDE score implied an increased possibility of tumor immune escape, indicating lower benefits from immune checkpoint inhibitor (ICI) therapy for patients (Jiang et al., 2018). Our findings indicated a higher TIDE score in the high-risk group, which suggested that immunotherapy might be less effective in this group of patients. In addition, we calculated the sensitivity of different risk groups to PCa chemotherapeutic agents using drug sensitivity analysis. Clinical trials of commonly used chemotherapy drugs for PCa have shown that using vinblastine or paclitaxel in combination with estramustine can treat patients with locally advanced or hormone-refractory PCa (Hudes et al., 1992; Zelefsky et al., 2000; Kelly et al., 2001; Urakami et al., 2002). The combination of docetaxel with androgen deprivation therapy (ADT) increased survival for those with metastatic hormone-sensitive PCa (Kyriakopoulos et al., 2018). Extensive clinical trials were established the therapeutic efficacy of agents such as rapamycin (Armstrong et al., 2010), sepantronium bromide (YM155) (Tolcher et al., 2012), and mitoxantrone (de Bono et al., 2010) in the treatment of PCa. In summary, the analysis of chemotherapeutic drug sensitivity offered new theoretical support for the clinical pharmacological management of PCa potentially.

There are limitations for this study. Firstly, the clinical cohort of all PCa cases in this study was obtained from public databases, and the sample size was limited. Future work will include larger clinical samples to enhance the accuracy of the results. Secondly, the outcomes of this study lacked validation through *in vitro* experiments. We will verify the expression patterns of these four key genes in the clinical samples using qPCR and immunohistochemistry, thereby increasing the reliability of the conclusions. Lastly, the potential mechanisms by which the four anoikis-related genes regulated the prognosis of PCa patients are required further investigation.

## 5 Conclusion

In conclusion, we developed a novel model incorporating four ARGs for predicting the risk of BCR in PCa. Our results offered a glimpse into the molecular and immunological characteristics of ARGs in PCa. In addition, we did a preliminary assessment of immunotherapy response and chemotherapeutic drug sensitivity in PCa patients from various risk groups. Collectively, this study potentially offered vital guidance for predicting BCR events in PCa.

## Data Availability

The original contributions presented in the study are included in the article/Supplementary Material, further inquiries can be directed to the corresponding authors.

## Supplementary material

SUPPLEMENTARY FIGURE S1 Construction and validation of risk model risk model. **(A)** Multivariate Cox regression analysis of the ARGs. **(B)** The risk score distribution and BCR status of PCa patients in the training set. **(C)** K‐M curves of BCR outcome between high‐ and low‐risk score groups in the training set. **(D)** ROC curves for BCR-free predictive performance at 3‐, 5‐, and 8-years in the training set. **(E‐F)** The boxplot and risk heatmap of the prognostic ARGsin the training set.

SUPPLEMENTARY FIGURE S2 The BCR-free rate of patients with PCa in the low- and high-risk group among the **(A)** Age > 60 ; **(B)** Gleason score > 7; **(C)** Gleason score <= 7 ; **(D)** N1; **(E)** T2; **(F)** T3/T4 .

SUPPLEMENTARY FIGURE S3 Validation of risk model based on GEO cohorts. The multivariate Cox regression analysis in two cohorts **(A)** GSE70770 **(B)** GSE46602.
