# Supplementary figures and images for: Identification of anoikis-related gene signatures and construction of the prognosis model in prostate cancer
Source: Front Pharmacol. 2024 Jun 18;15:1383304. doi: 10.3389/fphar.2024.1383304 (PMC11217483; doi:10.3389/fphar.2024.1383304)

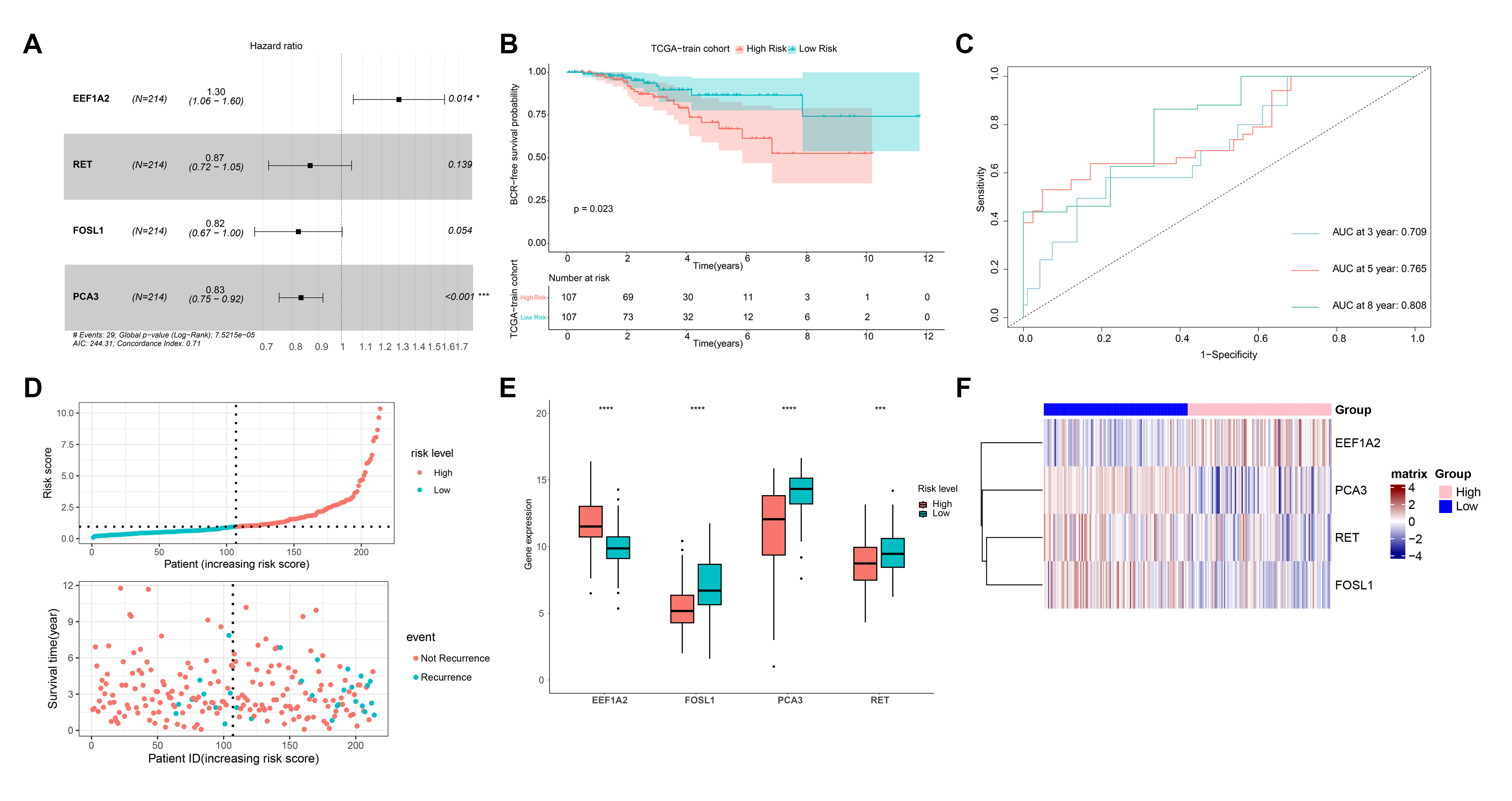

Supplement: Supplementary file 1 [file Image3.TIF]

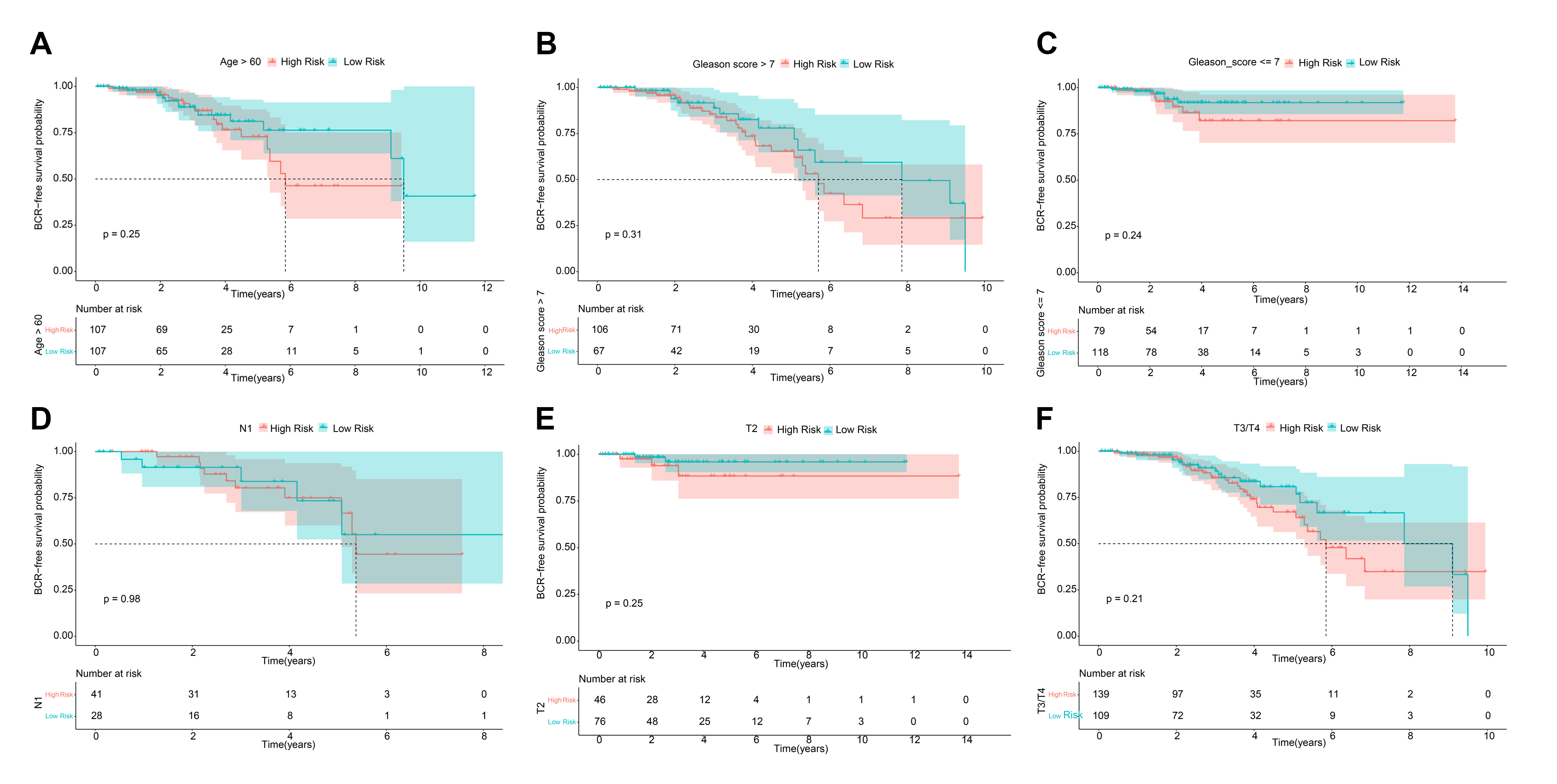

Supplement: Supplementary file 2 [file Image2.TIF]

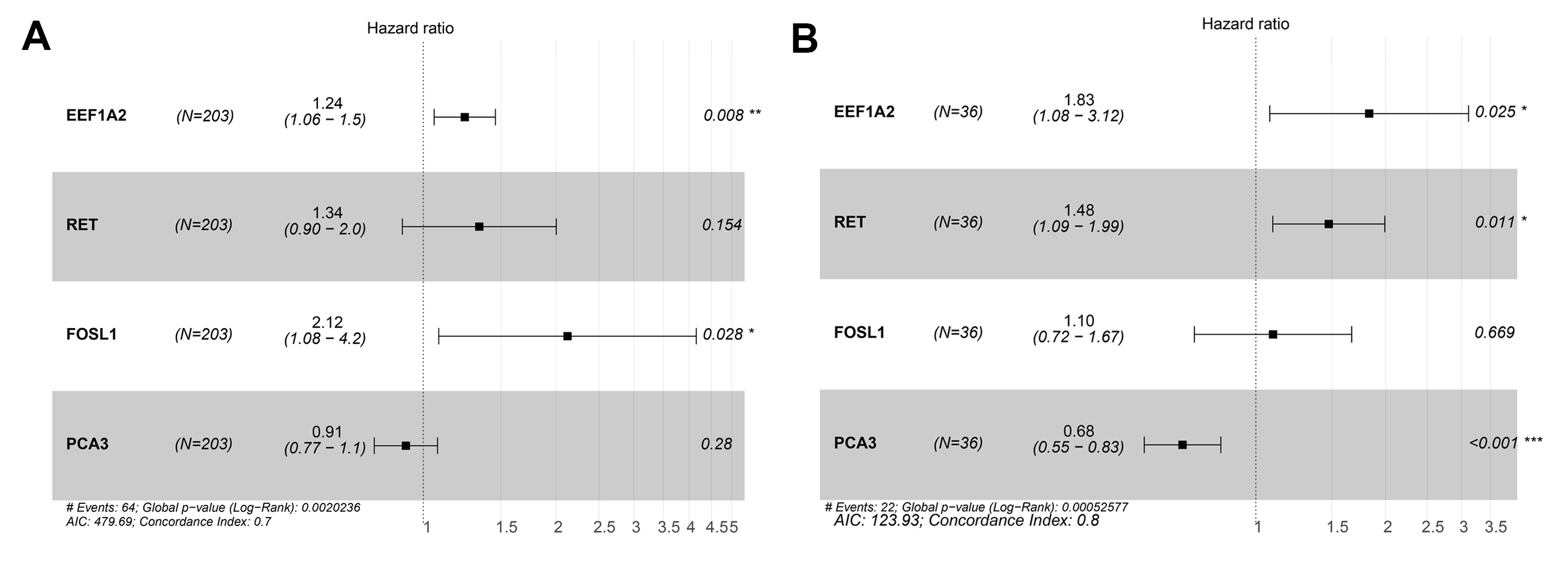

Supplement: Supplementary file 3 [file Image1.TIF]
